# Identification of a novel multifunctional oxidosqualene cyclase from *Zea mays* sheds light on the biosynthetic pathway of three pentacyclic triterpenoids

**DOI:** 10.1016/j.synbio.2022.08.004

**Published:** 2022-08-25

**Authors:** Zhenjun Fan, Yan Wang, Chengshuai Yang, Zhihua Zhou, Pingping Wang, Xing Yan

**Affiliations:** a CAS-Key Laboratory of Synthetic Biology, CAS Center for Excellence in Molecular Plant Sciences, Chinese Academy of Sciences, Shanghai, 200032, China; b University of Chinese Academy of Sciences, Beijing, 100049, China

**Keywords:** Oxidosqualene cyclase, *Zea mays*, Hop-17(21)-en-3-ol, Hopenol B, Simiarenol

## Abstract

*Zea mays* (maize) is an important agricultural crop that produces a variety of valuable terpenoids, including several triterpenoids. However, no oxidosqualene cyclase (OSC) enzymes, which catalyze the first step in triterpenoid biosynthesis, have been identified in maize. Here, we identified a novel OSC (*ZmOSC1*) in maize using a combination of genomic mining and phylogenetic analyses*.* To functionally characterize the candidate OSC, we constructed a yeast strain that produced high levels of 2,3-oxidosqualene. When ZmOSC1 was expressed in this strain, three compounds were detected and identified as hop-17(21)-en-3-ol, hopenol B and simiarenol, respectively. For their biosynthesis, we proposed a potential cyclization mechanism catalyzed by ZmOSC1 via the generation of a dammarenyl cation, followed by sequential cationic ring expansion, cyclization, cationic migration and further proton elimination. This study discovered and characterized an OSC from maize for the first time, and laid a foundation to produce three bioactive pentacyclic triterpenes, hop-17(21)-en-3-ol, hopenol B and simiarenol, using synthetic biology approaches.

## 1 Introduction

Triterpenoids are natural plant products that play an important role in mediating interactions between plants and the environment, and are widely used in medical, food, and cosmetic industries, as well as for industrial raw materials [1]. Oxidosqualene cyclases (OSCs) catalyze the cyclization of 2,3-oxidosqualene to form various triterpene scaffolds. These scaffolds are further modified by enzymes such as cytochrome P450s, acyltransferases and glycosyltransferase, which combine to generate tremendous structural diversity.

More than 20,000 triterpenoids with at least 100 different triterpenoid scaffolds have been identified in plants [3]. However, only ∼150 OSCs producing 50 different scaffolds have been characterized, most of which are of dicotyledonous origin [[1], [2]]. At present, only eleven monocotyledonous OSCs have been reported, among which seven are from rice (*Oryza sativa*) including isoarborinol synthase [4], parkeol synthase [5], cycloartenol synthases (OSC2 [5], CS1 [6], CsOSC1 [7], CAS1) and a multifunctional synthase that generates achilleol B as its main product [5]. Other monocotyledonous OSCs include CsOSC2, a multifunctional synthase from crape ginger (*Costus speciosus*) that produces lupeol, *β*-amyrin, germanicol and other triterpenoids [7], and AsbAS1, a *β*-amyrin synthase from *Avena strigosa* (oat) [6,8]. Recently, another two OSC genes were identified from oat, including hopenol B synthase AsHS1 and hop-17(21)-en-3-ol synthase AsHS2 and their mechanism affecting hydride shifting and deprotonation catalytic mechanism was revealed [9].

Previous phylogenetic analyses revealed that as well as the highly conversed cycloartenol synthase, the monocotyledonous OSCs were characterized to date cluster independently to the dicotyledonous OSCs, in contrast to typical function-related clustering [10]. For example, *β*-amyrin synthase of the monocotyledonous plant oat (AsbAS1) [6] is distinct from dicotyledonous *β*-amyrin synthases [[10], [11], [12], [13], [14], [15], [16], [17], [18], [19], [20]], which form a large independent phylogenetic group. The evolutionary relationships between monocotyledonous OSCs differ from those of dicotyledonous OSCs; thus, characterizing novel monocotyledonous OSCs is of great interest. *Zea mays* (maize), one of the most important agricultural crops worldwide, produces a variety of important triterpenoids such as α-amyrin, *β*-amyrin and cycloartenol [21]. However, no OSCs have been identified in maize.

In this study, we cloned and functionally characterized the OSC gene *ZmOSC1* from maize*.* ZmOSC1 forms a dammarane triterpenoid cation from 2,3-oxidosqualene via a chair-chair-chair (CCC) configuration, which can be further converted to three triterpenoid end products, hop-17(21)-en-3-ol, hopenol B and simiarenol. This is the first OSC from maize to be characterized, expanding our knowledge of triterpenoid biosynthesis in monocotyledonous plants. By characterizing ZmOSC1 in yeast, we have elucidated the biosynthetic pathways of hop-17(21)-en-3-ol, hopenol B and simiarenol, which exhibit various bioactivities. Therefore, the results of this study have paved the way for future production of these valuable triterpenoids using synthetic biology approaches.

## 2 Materials and methods

### 2.1 Materials, strains and reagents

The *Zea mays* cultivars were grown at Shanghai farm under natural light conditions. The *Saccharomyces cerevisiae* strain CEN.PK2–1C was obtained from EUROSCARF (Oberursel, Germany). The genes *ERG10, ERG13, ERG12, ERG8, ERG19, IDI*, and *tHMG1* were introduced into CEN.PK2–1C yeast cells to convert acetyl-CoA into isopentyl pyrophosphate and dimethylallyl pyrophosphate. Then, *ERG1, ERG20* and *ERG9* genes were introduced into the modified yeast to produce 2,3-oxidosqualene, which is the direct precursor of triterpenoid compounds. The production of 2,3-oxidosqualene was augmented by metabolic flow optimization to generate a triterpene-producing chassis strain. The *ZmOSC1* gene was inserted into the plasmid pESC-leu2d [22] and transformed into the ZFctr to generate *S. cerevisiae* strain ZF01. ZF01 was cultured on SCO plates (6.7 g/L yeast nitrogen base without amino acids, 20 g/L glucose) containing lysine, histidine and uracil.

### 2.2 Bioinformatics analysis

Proteins encoded by maize genome were download from PlantCyc (PMN11). A public Hidden Markov Model (PF13243, http://pfam.xfam.org/) was used to predict OSCs of maize [23]. All the hits with amino acid sequence length greater than 600 were selected for functional identification. The amino acid sequences of the candidate triterpene synthases were analyzed using MAFFT software and the phylogenetic tree was constructed using the neighbor–joining method with Geneious R8 software, with 1000 bootstrap replicates. Accession numbers of the related triterpene synthase sequences were listed in the Supplementary Table S4.

### 2.3 Cloning of OSC genes

Candidate OSC genes of *ZmOSC1-8* were deposited in the Registry and database of bioparts for synthetic biology (https://www.biosino.org/npbiosys/) under accession nos. OENC366048-OENC366055. They were selected and cloned from cDNA extracted from *Zea mays* leaf tissue using the PrimeScript RT reagent kit with gDNA Eraser (Takara, Dalian, China). The open reading frame (ORF) of each gene was PCR-amplified using the primers shown in the supplementary data. The PCR products were cloned into pEASY-Blunt Simple Cloning Vector (TransGen, Beijing, China) for sequencing.

### 2.4 Heterologous expression of ZmOSC1 in yeast

To functionally characterize the ZmOSCs, the cloned sequences were co-transformed with the plasmid pESC-leu2d (after *SmaI* digestion) into the ZFctr to generate strains ZF01–ZF04. The yeast strains were grown in SCO medium composed of 6.7 g/L yeast nitrogen base without amino acids (Sinopharm Chemical, Shanghai, China), 20 g/L glucose, 0.1 g/L lysine, 0.1 g/L histidine and 0.1 g/L uracil.

Next, the engineered yeast strains were fermented in shake flasks. Individual clones were inoculated into 10 mL of minimal medium with 1‰ 150 mM copper sulfate, and cultivated at 30 °C with shaking at 250 rpm for 4 days. One milliliter of fermentation liquid was harvested, disrupted and extracted with 20% potassium hydroxide (w/v) and 50% ethanol (v/v), and then extracted twice with a similar volume of n-hexane [24]. The extract was evaporated and resuspended in 50 μL ethyl alcohol for high-performance liquid chromatography (HPLC) analysis.

### 2.5 HPLC analysis

HPLC analysis was performed using a Shimadzu LC 20A system (Shimadzu, Kyoto, Japan) equipped with a LC20ADXR pump, autoplate, binary pump and a diode array detector. To analyze the products extracted from strains ZF01-ZF04, chromatographic separation was performed at 35 °C with a Welch Boltimate™ C_18_ (2.7 μm, 2.1 × 100 mm) column. The gradient elution system consisted of water (A) and acetonitrile (B). The enzyme reaction extract was separated using the following gradient: 0–2 min (45% B), 2–15 min (45–100% B), 15–27 min (100% B) and 27–30 min (45% B). The flow rate was kept at 0.45 mL/min. The products were monitored at 203 nm absorbance. Semi-preparative HPLC was performed using the LC 20A system, fitted with a SinoChrom ODS-BP column (5 μm, 10 × 250 mm) and with a 100% acetonitrile mobile phase. The flow rate was maintained at 2.5 mL/min, and the products were monitored at 203 nm absorbance.

### 2.6 Liquid chromatography-atmospheric pressure chemical ionization mass spectrometry (LC-APCIMS) analysis

Liquid chromatography-atmospheric pressure chemical ionization mass spectrometry (LC-APCIMS) analysis was performed using an UHPLC1290II-MS/APCI QT0F6545 (Agilent, Santa Clara, CA, USA) equipped with a Welch Boltimate™ C_18_ (2.7 μm, 2.1 × 100 mm) column. The mobile phase comprised water (A) and acetonitrile (B), and the flow rate was maintained at 0.35 mL/min. The gradient program was as follows: 0–2 min (45% B), 2–12 min (45–100% B), 12–24 min (100% B), and 24–28 min (45% B). The LC-APCIMS data were acquired using an orthogonal acceleration time-of-flight mass spectrometer (Agilent) in positive ionization mode.

### 2.7 Nuclear magnetic resonance (NMR) analysis

NMR spectra were recorded on the Bruker Advance III 500 instrument (^1^H: 500 MHz, ^13^C: 125 MHz; Bruker, Billerica, MA, USA), with chemical shift values expressed as ppm relative to tetramethylsilane (*δ_H_* 0.00 ppm) or residual chloroform (*δ_H_* 7.26 and *δ_C_* 77.16 ppm) as a standard.

## 3 Results and discussion

### 3.1 Prediction and cloning of novel OSCs from *Zea mays*

**Fig. 1:**
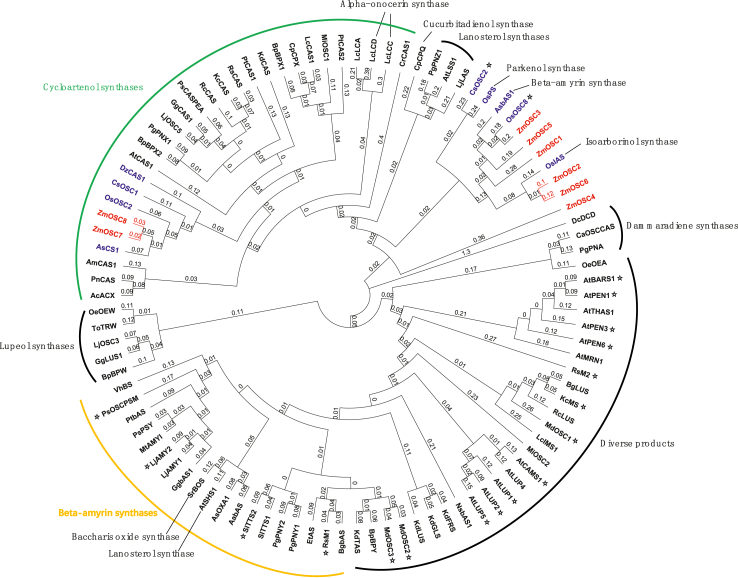
Phylogenetic analysis of oxidosqualene cyclases (OSCs) from *Zea mays* and other OCSs from diverse plant species. The tree was constructed via the neighbor-joining algorithm. Accession numbers are provided in Table S4. Monocotyledonous OSCs are marked in blue, and *Zea mays* OSCs (ZmOSCs) in red. The other OSCs derived from dicotyledonous are shown in black. Cycloartenol synthases are shown in green; *β*-amyrin synthases are shown in orange. Multifunctional OSCs are marked with an asterisk.

A total of eight candidate OSCs of maize, namely *ZmOSC1–8*, were predicted (Table S1). To further analyze the relationships between the candidate OSCs and other known plant OSCs, we generated a phylogenetic tree (Fig. 1). Consistent with previous reports [1], monocotyledonous OSCs including the six candidate OSCs, ZmOSC1–6, grouped into a single cluster. This cluster was distinct from dicotyledonous OSCs, which clustered into a different group and exhibited a strong functional relationship. ZmOSC7 and ZmOSC8 shared 87.22% and 86.82% sequence identities to the cycloartenol synthases OsOSC2 and AsCS1, respectively, and clustered within the cycloartenol synthase clade. Because these two candidates were most likely to be cycloartenol synthases, we focused on characterizing ZmOSC1–6. Of the six OSC genes, four (*ZmOSC1–4*) were successfully cloned. Among them, ZmOSC1 had the lowest identity with identified OSCs (61.35%), and ZmOSC2, ZmOSC3 and ZmOSC4 were 77.81%, 64.39% and 77.98%, respectively.

### 3.2 Functional characterization of ZmOSCs

2,3-oxidosqualene is the precursor for most plant triterpenoids. Although *S. cerevisiae* has a native 2,3-oxidosqualene biosynthetic pathway, its production amount is low. To increase 2,3-oxidosqualene biosynthesis and yield in yeast, we constructed a yeast chassis strain with high-level 2,3-oxidosqualene production. Seven key genes of the mevalonate pathway, *ERG10, ERG13, ERG12, ERG8, ERG19, IDI* and *tHMG1*, catalyze the conversion of acetyl-CoA into isopentyl pyrophosphate and dimethylallyl pyrophosphate. These genes were introduced and overexpressed in the yeast strain CEN.PK2–1C. Then, the *ERG1, ERG20,* and *ERG9* genes were introduced to improve 2,3-oxidosqualene production. To reduce the consumption of 2,3-oxidosqualene via the formation of lanosterol and ergosterol, the expression of *ERG7* was down-regulated by replacing its native promoter with the Cu^2+^-repressed promoter *CTR3*, thus completing the construction of the chassis strain ZFctr. The production of 2,3-oxidosqualene was significantly higher in ZFctr than CEN.PK2–1C (Fig. S1).

**Fig. 2:**
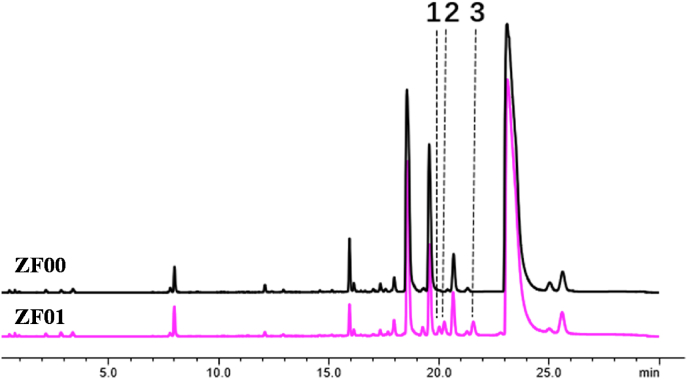
HPLC analysis (UV 203 nm) of metabolic products extracted from ZF00 and ZF01. Peaks **1**–**3** represent the three triterpenes produced by ZF01 *in vivo*.

To functionally validate the ZmOSCs, the ORFs of the four OSC candidates were ligated into the yeast expression plasmid pESC-leu2d, to generate plasmids pESC-leu2d-ZmOSC1–4. These plasmids were transformed into the yeast chassis strain ZFctr, and the resulting recombinant strains were named ZF01–ZF04. Similarly, the control strain ZF00 was constructed by transforming ZFctr with empty pESC-leu2d plasmid. After 4 days of fermentation in shake flasks, the metabolites produced by ZF01–ZF04 and ZF00 were extracted and analyzed by HPLC. No peaks representing novel triterpenoids were present in the spectra of strains ZF03 and ZF04, using the spectrum of the control strain ZF00 for comparison. However, three new peaks were detected in strain ZF01 expressing ZmOSC1 (Fig. 2), and one new peak was present in strain ZF02 harboring the *ZmOSC2* gene (Fig. S2). The retention time of the newly appeared peak from ZmOSC2 was identical to the second new peak from ZmOSC1 (Fig. S2), ZmOSC2 likely produces a compound identical to one of these produced by ZmOSC1 and thus was not selected for further characterization.

### 3.3 Structural identification of triterpenoids produced by ZmOSC1

**Fig. 3:**
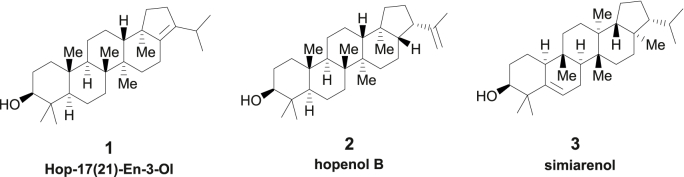
Structures of compounds **1**–**3** produced by ZF01.

The metabolites extracted from ZF01 and ZF00 were analyzed by LC-APCIMS (Fig. S3). Three new peaks with the same mass-to-charge ratio (*m/z* 409.3890 [M + H - H_2_O]^+^) were present in ZF01. To further examine the structure of the three triterpenoids produced by ZmOSC1, large-scale fermentation of ZF01 was performed, and the three triterpenoid products were purified and subjected to NMR analysis. **1** was produced *in vivo* by strain ZF01 from the common triterpenoid precursor, 2,3-oxidosqualene. LC-APCIMS (*m/z* 409.3886 [M + H - H_2_O]^+^, calculated (409.3890 [M + H - H_2_O]^+^) and ^1^H, ^13^C NMR spectra from both the present and previous studies suggested that **1** was hop-17(21)-en-3-ol (Fig. 3). Hop-17(21)-en-3-ol was isolated previously from *Quercus championi* [25], *Gentiana scabra* [26] and *Avena strigose,* which is component of surface wax in oat sheathes [9]. **2** was also produced *in vivo* by strain ZF01. Comparison of our LC-APCIMS (*m/z* 409.3886 [M + H - H_2_O]^+^, calculated 409.3890 [M + H - H_2_O]^+^) and ^1^H, ^13^C NMR spectra with those of previous studies suggested that the purified triterpenoid **2** was hopenol B (Fig. 3). Hopenol B has antioxidant, anti-inflammatory and anti-ulcerogenic activities [27,28]. It's an active ingredient in herbs that could protect the liver [29] or protect plants as onecomponent of surface wax in oat panicles [9].

Triterpenoid **3** was purified by semi-preparative HPLC following its production *in vivo* by strain ZF01. When compared to previous studies, our LC-APCIMS (*m/z* 409.3886 [M + H - H_2_O]^+^, calculated 409.3890 [M + H - H_2_O]^+^) and ^1^H, ^13^C NMR spectra suggested that **3** was simiarenol (Fig. 3). Simiarenol, which was previously isolated from three European *Euphorbia* species and other plants [30,31], has significant α-glucoside enzyme inhibition activity [32], which reduces postprandial hyperglycemia. Furthermore, simiarenol exhibits anti-leishmanial activities [33]. Leishmaniasis is a health problem worldwide, particularly in developing countries.

The three triterpenoid compounds isolated in this study, hop-17(21)-en-3-ol, hopenol B and simiarenol, have not previously been isolated from *Zea mays*. However, the identification of ZmOSC1 suggested that *Zea mays* might be able to produce these three triterpenoids, even under conditions in which their production is low or completely inhibited. The identification of ZmOSC1 has uncovered the biosynthetic pathways of hop-17(21)-en-3-ol, hopenol B and simiarenol, providing a means for large-scale synthesis of these triterpenoids and analysis of their biological activity. The role of these triterpenoids in maize pest resistance is unknown due to the low expression levels of the key biopart (*ZmOSC1*). Heterologous biosynthesis of these triterpenoids may also facilitate optimization of their metabolic pathway in maize, thus enhancing the resistance of maize to pests and diseases.

Recently, AsHS1 (hopenol B synthase) and AsHS2 (hop-17(21)-en-3-ol synthase) were also identified in oat. The amino acid sequences identity of ZmOSC1 towards AsHS2 and AsHS1 was 59.5% and 61.4%, respectively. Hopenol B synthase AsHS1 and hop-17(21)-en-3-ol synthase AsHS2 identified in oat can change their function through the mutation of key amino acid residues at sites 121, 410 and 722. ZmOSC1 was a mixed hopane triterpenoid synthase with three products, hop-17(21)-en-3-ol, hopenol B and simiarenol. Both ZmOSC1 and AsHS2 are isoleucine at site 121 with the same product hop-17(21)-en-3*β*-ol, while ZmOSC1, AsHS1 and AsHS2 are different at site 722. This is consistent with the conclusion that site 121 is more important than site 722 for hop-17(21)-en-3*β*-ol synthase [9]. Both ZmOSC1 and AsHS1 are tyrosine at site 410 with the same product, hopenol B, while AsHS1 is alanine. These results are helpful to reveal the relationship between the sequence and function of hopane triterpenoid synthases in different plants.

### 3.4 Proposed cyclization pathway of triterpene hop-17(21)-en-3-ol, hopenol B and simiarenol

**Fig. 4:**
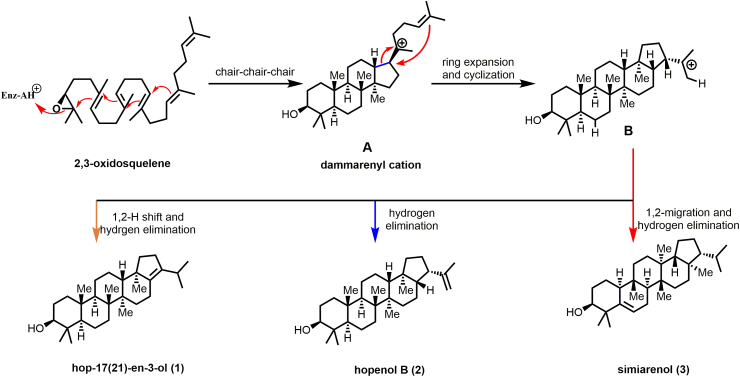
Proposed cyclization pathway of **1** (hop-17(21)-en-3-ol), **2** (hopenol B) and **3** (simiarenol).

OSCs are capable of enabling the formation of more complicated derivatives bearing four or five ring systems from the liner 2,3-oxidosqualene. However, the detailed mechanism of this process is not fully elucidated. In light of the experimental results and computational calculation reported in the known literatures [9,34,35], the formation of triterpenoid scaffolds might undergo sequential protonation, cyclization, migration, and finally vicinal hydrogen elimination or hydroxylation with water. Depending on conformational analysis of the resulted products, the molecular skeletons can be classified into two series: sterols and triterpenes. In the process of sterol biosynthesis, 2,3-oxidosqualene was cyclized via the chair-boat-chair conformation. In contrast, triterpene was obtained via the chair-chair-chair conformational cyclization [1]. We have investigated the stereochemistry of the cyclic products **1**–**3** generated by ZmOSC1, and identified the three cyclic products as triterpene compounds. Inspiring by the enzyme-catalyzed cyclization mechanism reported previously [34,35], we hypothesized that the dammarane triterpene cation intermediate **A** could be generated from 2,3-oxidosqualene by ZmOSC1 via a chair–chair–chair conformation cyclization, followed by sequential cationic ring expansion and further cyclization to afford intermediate **B** (Fig. 4). Finally, hopenol B (**2**) could be readily formed via proton elimination of the cation intermediate **B**. Moreover, Hop-17(21)-en-3-ol (**1**) and simiarenol (**3**) might be generated via a more complicated 1,2-cation migration and proton elimination process (Fig. S4).

## 4 Conclusions

Through genome mining and phylogenetic analysis, we discovered a novel triterpene synthase, ZmOSC1, in maize. The expression of ZmOSC1 in a 2,3-oxidosqualene producing yeast strain led to the accumulation of three bioactive pentacyclic triterpenes: hop-17(21)-en-3-ol, hopenol B and simiarenol. Our work not only provides insight into the biosynthesis of triterpenoids in maize, but also identified a novel biopart enabling the construction of cell factories that produce these valuable triterpenoids, in turn facilitating their large-scale production.

## CRediT authorship contribution statement

**Zhenjun Fan:** Conceptualization, Investigation, Writing – original draft. **Yan Wang:** Investigation, Writing – review & editing. **Chengshuai Yang:** Investigation. **Zhihua Zhou:** Conceptualization, Supervision, Project administration, Writing – review & editing. **Pingping Wang:** Investigation, Writing – review & editing, Supervision. **Xing Yan:** Conceptualization, Writing – review & editing, Supervision.

## References

[1] Thimmappa R., Geisler K., Louveau T., O'Maille P., Osbourn A. (2014). Triterpene biosynthesis in plants. Annu Rev Plant Biol.

[2] Wang J., Guo Y., Yin X., Wang X., Qi X., Xue Z. (2022). Diverse triterpene skeletons are derived from the expansion and divergent evolution of 2,3-oxidosqualene cyclases in plants. Crit Rev Biochem Mol Biol.

[3] Xu R., Fazio G.C., Matsuda S.P. (2004). On the origins of triterpenoid skeletal diversity. Phytochemistry.

[4] Xue Z., Duan L., Liu D., Guo J., Ge S., Dicks J. (2012). Divergent evolution of oxidosqualene cyclases in plants. New Phytol.

[5] Ito R., Mori K., Hashimoto I., Nakano C., Sato T., Hoshino T. (2011). Triterpene cyclases from oryza sativa l.: cycloartenol, parkeol and achilleol b synthases. Org Lett.

[6] Haralampidis K., Bryan G., Qi X., Papadopoulou K., Bakht S., Melton R. (2001). A new class of oxidosqualene cyclases directs synthesis of antimicrobial phytoprotectants in monocots. Proc Natl Acad Sci U S A.

[7] Kawano N., Ichinose K., Ebizuka Y. (2002). Molecular cloning and functional expression of cdnas encoding oxidosqualene cyclases from costus speciosus. Biol Pharm Bull.

[8] Qi X., Bakht S., Leggett M., Maxwell C., Melton R., Osbourn A. (2004). A gene cluster for secondary metabolism in oat: implications for the evolution of metabolic diversity in plants. Proc Natl Acad Sci U S A.

[9] Liang M., Zhang F., Xu J., Wang X., Wu R., Xue Z. (2022). A conserved mechanism affecting hydride shifting and deprotonation in the synthesis of hopane triterpenes as compositions of wax in oat. Proc Natl Acad Sci U S A.

[10] Meesapyodsuk D., Balsevich J., Reed D.W., Covello P.S. (2007). Saponin biosynthesis in saponaria vaccaria. Cdnas encoding beta-amyrin synthase and a triterpene carboxylic acid glucosyltransferase. Plant Physiol.

[11] Morita M., Shibuya M., Lee M.S., Sankawa U., Ebizuka Y. (1997). Molecular cloning of pea cdna encoding cycloartenol synthase and its functional expression in yeast. Biol. Pharmaceut. Bull..

[12] Sawai S., Shindo T., Sato S., Kaneko T., Tabata S., Ayabe S. (2006). Functional and structural analysis of genes encoding oxidosqualene cyclases of lotus japonicus. Plant Sci.

[13] Iturbe-Ormaetxe I., Haralampidis K., Papadopoulou K., Osbourn A.E. (2003). Molecular cloning and characterization of triterpene synthases from medicago truncatula and lotus japonicus. Plant Mol Biol.

[14] Hayashi H., Huang P.Y., Kirakosyan A., Inoue K., Hiraoka N., Ikeshiro Y. (2001). Cloning and characterization of a cdna encoding beta-amyrin synthase involved in glycyrrhizin and soyasaponin biosyntheses in licorice. Biol. Pharmaceut. Bull..

[15] Cammareri M., Consiglio M.F., Pecchia P., Corea G., Lanzotti V., Ibeas J.I. (2008). Molecular characterization of beta-amyrin synthase from aster sedifolius l. And triterpenoid saponin analysis. Plant Sci.

[16] Kirby J., Romanini D.W., Paradise E.M., Keasling J.D. (2008). Engineering triterpene production in saccharomyces cerevisiae-beta-amyrin synthase from artemisia annua. FEBS J.

[17] Kajikawa M., Yamato K.T., Fukuzawa H., Sakai Y., Uchida H., Ohyama K. (2005). Cloning and characterization of a cdna encoding beta-amyrin synthase from petroleum plant euphorbia tirucalli l. Phytochemistry.

[18] Wang Z.H., Guhling O., Yao R.N., Li F.L., Yeats T.H., Rose J.K.C. (2011). Two oxidosqualene cyclases responsible for biosynthesis of tomato fruit cuticular triterpenoids. Plant Physiol.

[19] Basyuni M., Oku H., Inafuku M., Baba S., Iwasaki H., Oshiro K. (2006). Molecular cloning and functional expression of a multifunctional triterpene synthase cdna from a mangrove species kandelia candel (l.) druce. Phytochemistry.

[20] Kushiro T., Shibuya M., Ebizuka Y. (1998). Beta-amyrin synthase - cloning of oxidosqualene cyclase that catalyzes the formation of the most popular triterpene among higher plants. Eur J Biochem.

[21] Guo D.A., Venkatramesh M., Nes W.D. (1995). Developmental regulation of sterol biosynthesis in zea-mays. Lipids.

[22] Ro D.K., Ouellet M., Paradise E.M., Burd H., Eng D., Paddon C.J. (2008). Induction of multiple pleiotropic drug resistance genes in yeast engineered to produce an increased level of anti-malarial drug precursor, artemisinic acid. BMC Biotechnol.

[23] Finn R.D., Mistry J., Schuster-Bockler B., Griffiths-Jones S., Hollich V., Lassmann T. (2006). Pfam: clans, web tools and services. Nucleic Acids Res.

[24] Itkin M., Davidovich-Rikanati R., Cohen S., Portnoy V., Doron-Faigenboim A., Oren E. (2016). The biosynthetic pathway of the nonsugar, high-intensity sweetener mogroside v from siraitia grosvenorii. Proc Natl Acad Sci U S A.

[25] Arthur H.R., Lam C.N., Szeto S.K., Hui W.H. (1964). Examination of quercus championi of Hong Kong. Aust J Chem.

[26] Kakuda R., Iijima T., Yaoita Y., Machida K., Kikuchi M. (2002). Triterpenoids from gentiana scabra. Phytochemistry.

[27] Okeleye B.I., Mkwetshana N.T., Ndip R.N. (2013). Evaluation of the antibacterial and antifungal potential of peltophorum africanum: toxicological effect on human chang liver cell line. Sci World J.

[28] Bedi O., Bijjem K.R.V., Kumar P., Gauttam V. (2016). Herbal induced hepatoprotection and hepatotoxicity: a critical review. Indian J Physiol Pharmacol.

[29] Jaishree V., Badami S., Krishnamurthy P.T. (2010). Antioxidant and hepatoprotective effect of the ethyl acetate extract of enicostemma axillare (lam). Raynal against ccl4-induced liver injury in rats. Indian J Exp Biol.

[30] Kwon H.C., Choi S.U., Lee K.R. (2001). Phytochemical constituents of artemisia stolonifera. Arch Pharm Res (Seoul).

[31] Csupor-Loffler B., Hajdu Z., Zupko I., Molnar J., Forgo P., Vasas A. (2011). Antiproliferative constituents of the roots of conyza canadensis. Planta Med.

[32] Anjum S., Asif M., Zia K., Jahan B., Ashraf M., Hussain S. (2020). Biological and phytochemical studies on capparis decidua (forssk) edgew from cholistan desert. Nat Prod Res.

[33] Amin E., Moawad A., Hassan H. (2017). Biologically-guided isolation of leishmanicidal secondary metabolites from euphorbia peplus l. Saudi Pharmaceut J.

[34] Wendt K.U., Schulz G.E., Corey E.J., Liu D.R. (2000). Enzyme mechanisms for polycyclic triterpene formation. Angew Chem Int Ed Engl.

[35] Corey E.J., Virgil S.C. (1991). An experimental demonstration of the stereochemistry of enzymic cyclization of 2,3-oxidosqualene to the protosterol system, forerunner of lanosterol and cholesterol. J Am Chem Soc.

