# Supplementary material for: Identification of a novel multifunctional oxidosqualene cyclase from Zea mays sheds light on the biosynthetic pathway of three pentacyclic triterpenoids
Source: Synth Syst Biotechnol. 2022 Aug 25;7(4):1167–72. doi: 10.1016/j.synbio.2022.08.004 (PMC9467860; doi:10.1016/j.synbio.2022.08.004)
Supplement: Multimedia component 1 [file mmc1.docx]

**Supplementary Information for:**

Identification of a novel multifunctional Oxidosqualene cyclase from *Zea mays* sheds light on the biosynthetic pathway of three pentacyclic triterpenoids

Zhenjun Fan ^a, b, 1^, Yan Wang ^a, 1^, Chengshuai Yang ^a^, Zhihua Zhou ^a, b^, Pingping Wang ^a, *^, Xing Yan ^a, b, *^

^a^ CAS-Key Laboratory of Synthetic Biology, CAS Center for Excellence in Molecular Plant Sciences, Chinese Academy of Sciences, Shanghai, 200032, China

^b^ University of Chinese Academy of Sciences, Beijing, 100049, China

^1^ These authors contributed equally to this work.

^*^Corresponding authors: Xing Yan (yanxing@cemps.ac.cn); Pingping Wang (ppwang@cemps.ac.cn)

## 5. Appendix- Supplementary materials

## 5.1 Supplemental File

**File S1.** NMR data of **1** (hop-17(21)-en-3-ol), **2** (hopenol B) and **3** (simiarenol)

## 5.2 Supplemental Figures


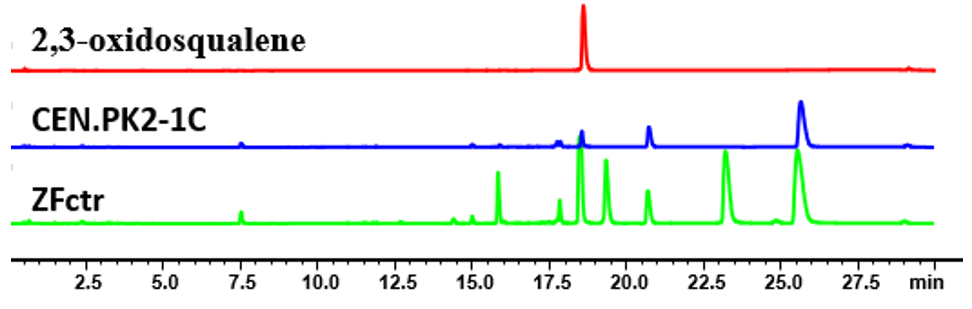


**Figure S1. 2,3-oxidosqualene in chassis strain CEN.PK2-1C and ZFctr by HPLC.** HPLC analysis of 2,3-oxidosqualene from original strain CEN.PK2-1C and enhanced strain ZFctr.

**
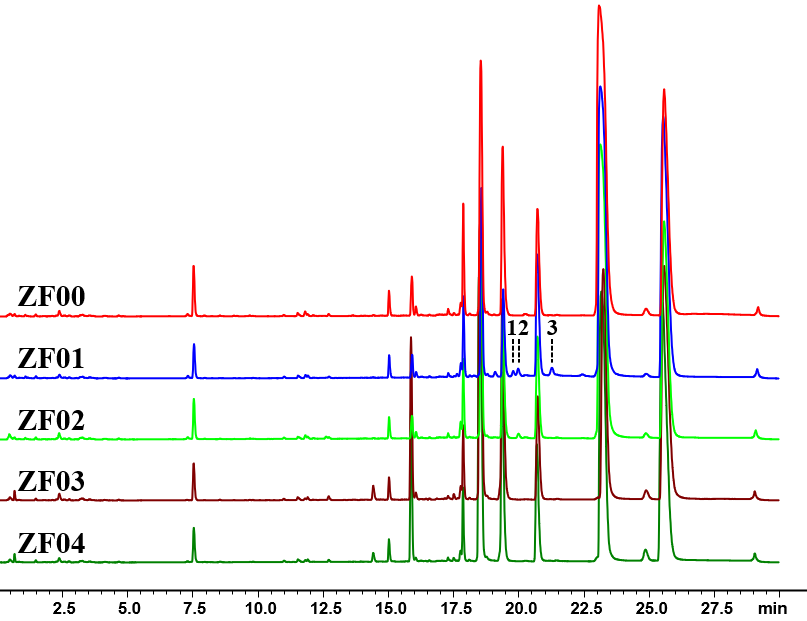
**

**Figure S2.** **Functional identification of ZF00-ZF04 by HPLC.** HPLC analysis of products from ZF00 to ZF04. The control strain ZF00 was constructed with the empty vector pESC-leu2d, and ZF01-ZF04 with pESC-leu2d-ZmOSC1-4. Peak **1**, **2** and **3** represent three products from ZF01 *in vivo*.


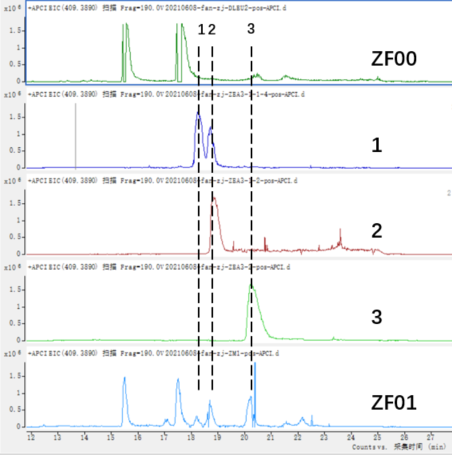


**Figure S3. LC-APCIMS analysis of products from ZF00-ZF01 and purified** **1**, **2** and **3.** Ion chromatograms of m/z 409.3890 were extracted. Peak **1,** hop-17(21)-en-3-ol, peak **2,** hopenol B and peak **3,** simiarenol.

**Figure S4. The proposed electron-transfer diagram for the triterpenes generated from intermediate B.** Hopenol B (**2**) could be formed via proton elimination of the cation intermediate **B**. Hop-17(21)-en-3-ol (**1**) and simiarenol (**3**) might be generated via 1,2-cation migration and proton elimination process.

**A**

**
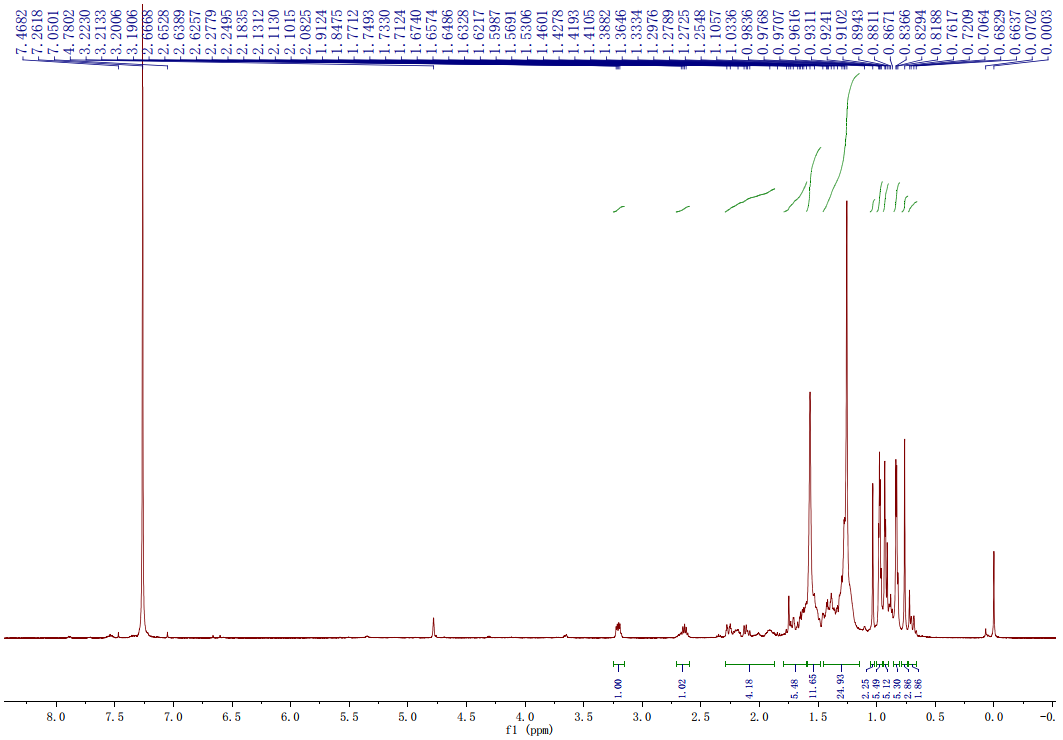
**

**B**

**
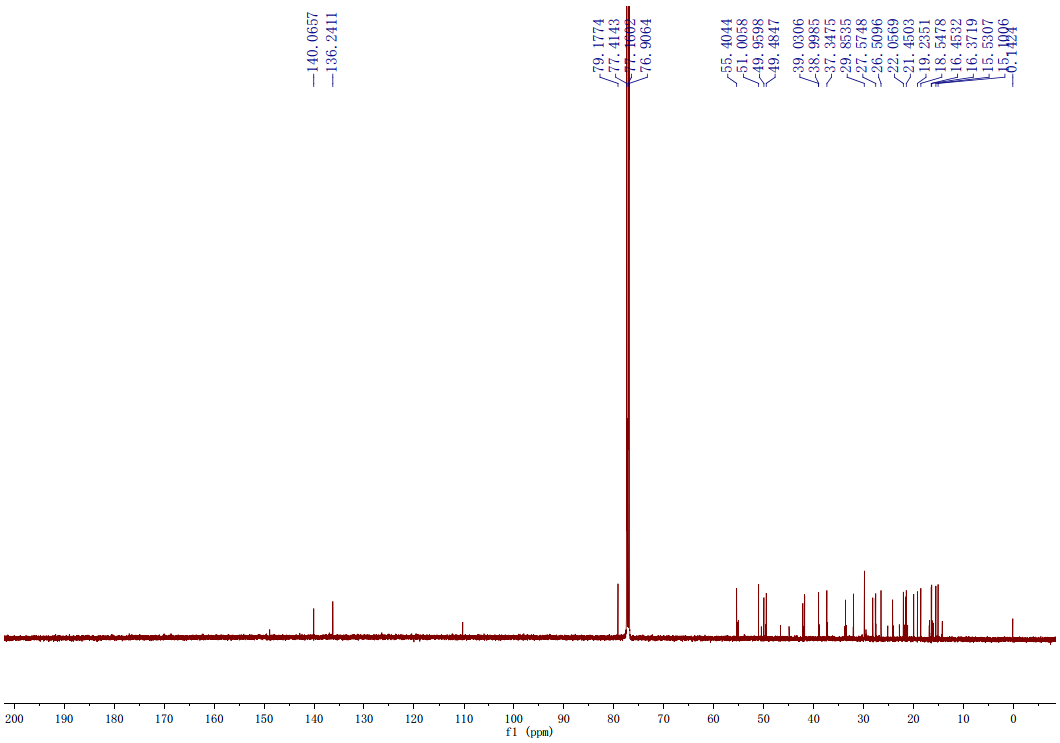
**

**C**

**
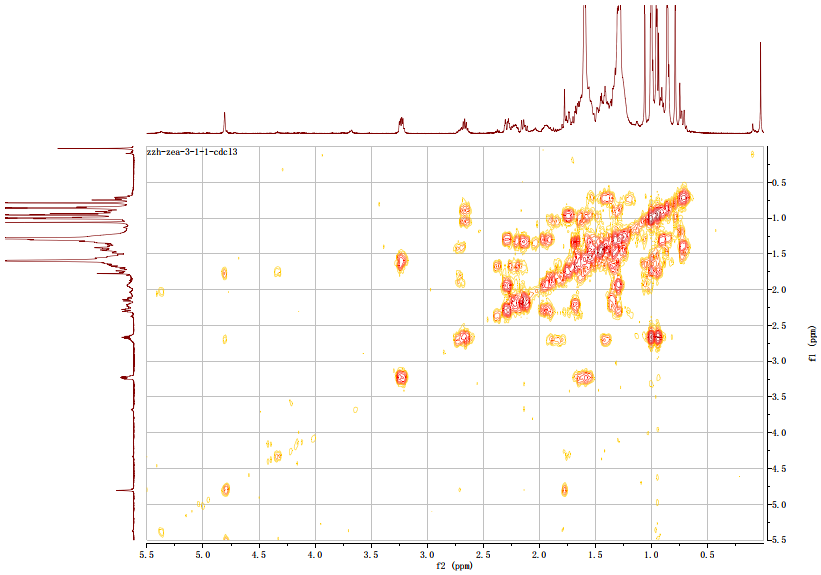
**

**D**

**
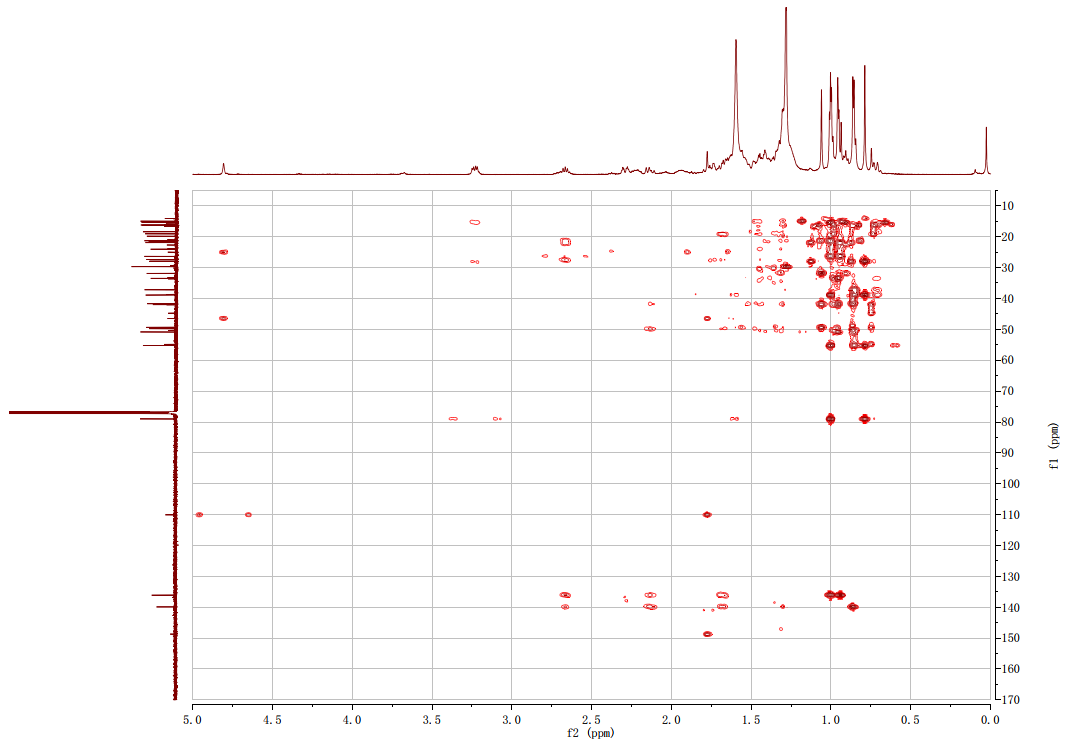
**

**E**

**
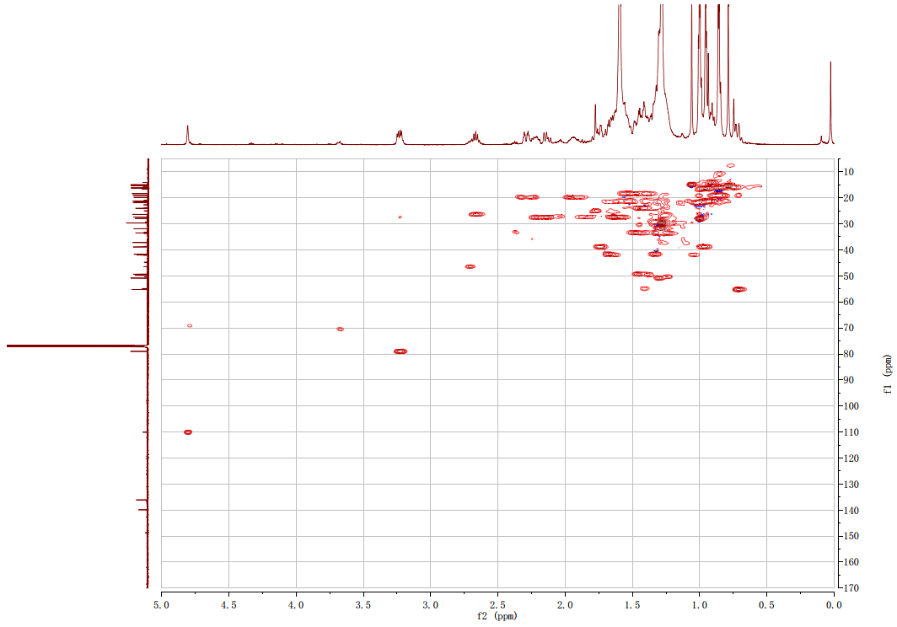
**

**F**

**
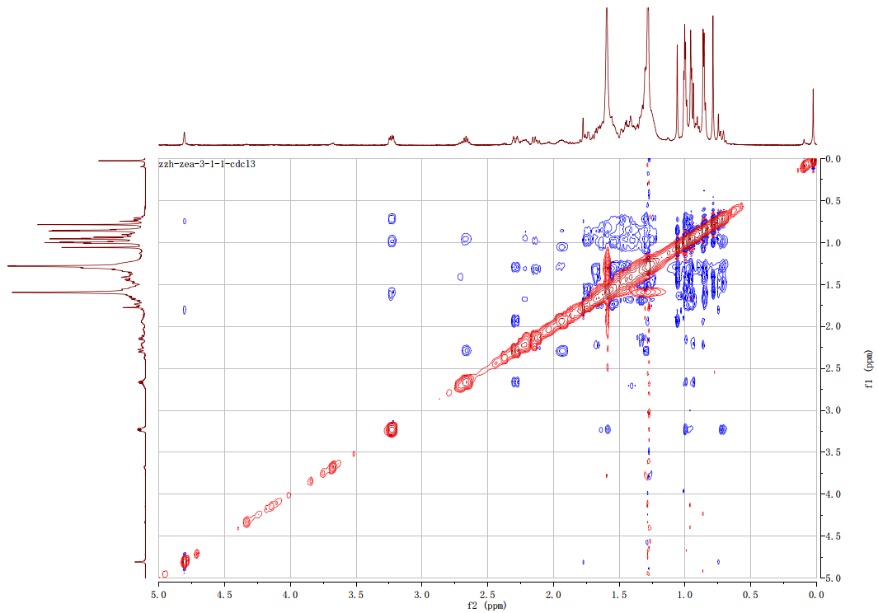
**

**G**

**
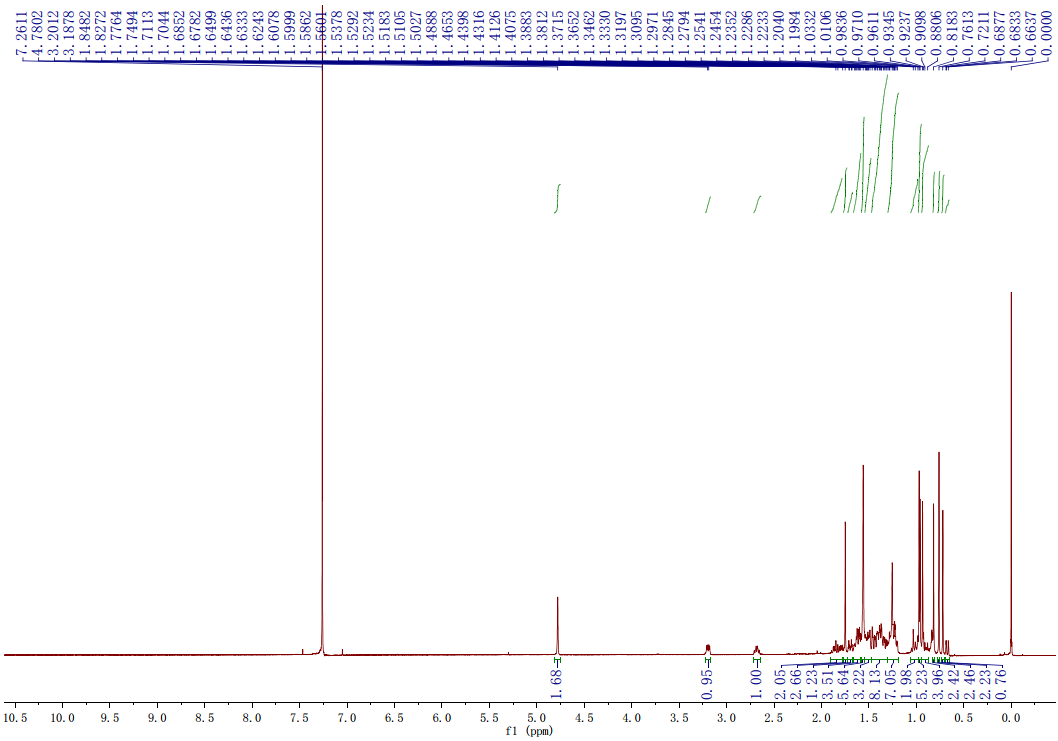
**

**H**

**
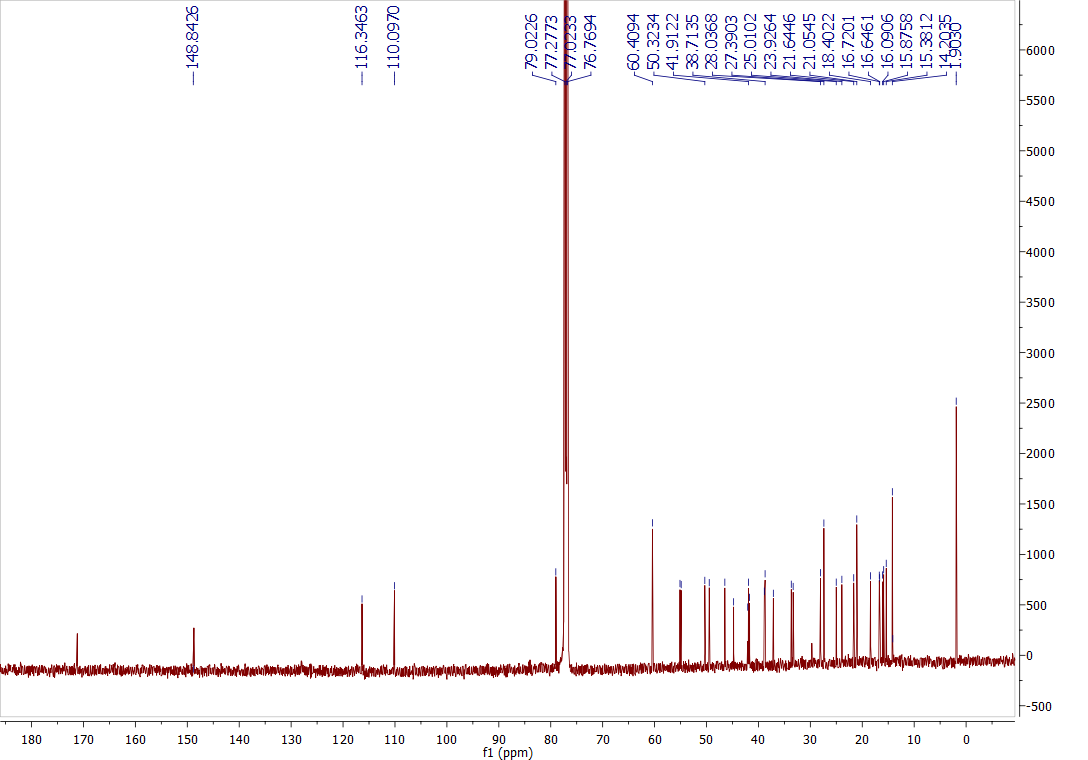
**

**I**

**
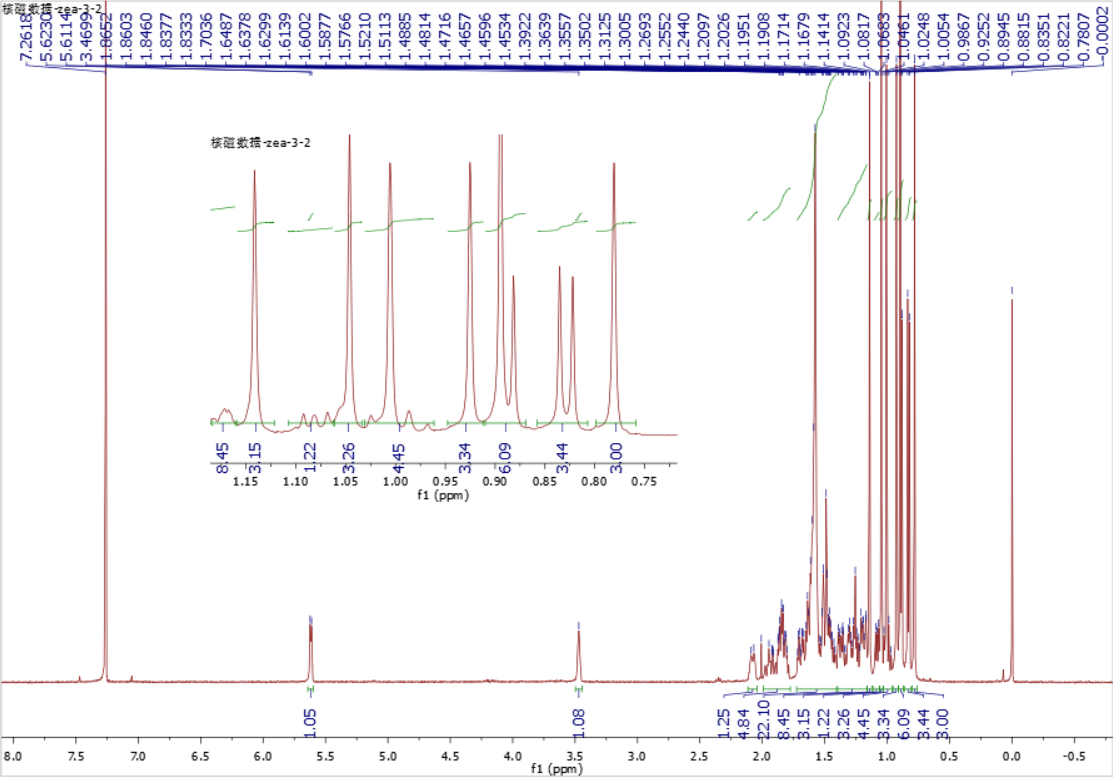
**

**J**

**
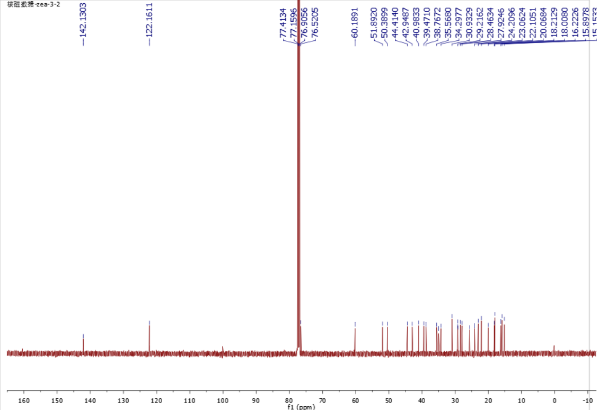

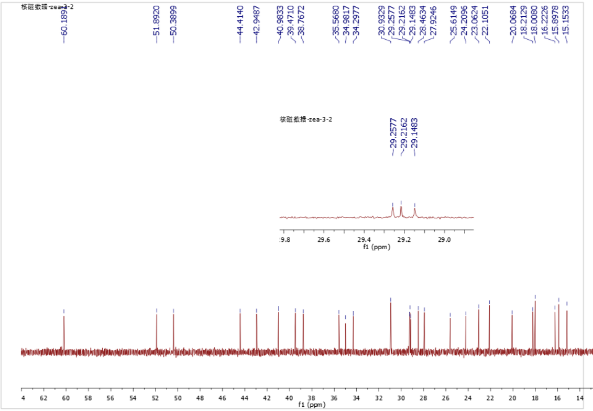
**

**Figure S5.** NMR spectral data of **1** (hop-17(21)-en-3-ol), **2** (hopenol B) and **3** (simiarenol), recorded in CDCl_3_.

**A.** ^1^H-NMR spectrum of **1**.

**B.** ^13^C-NMR spectrum of **1**.

**C.** COSY spectrum of **1**.

**D.** HMBC spectrum of **1**.

**E.** HSQC spectrum of **1**.

**F.** NOE spectrum of **1**.

**G.** ^1^H-NMR spectrum of **2**.

**H.** ^13^C-NMR spectrum of **2**.

**I.** ^1^H-NMR spectrum of **3**.

**J.** ^13^C-NMR spectrum of **3**.

## 5.3 Supplemental Tables

**Table S1.** **Candidate triterpene synthases in *Zea mays***

| **Gene name** | **Accession number** | **Identity(%)** | **The nearest identified OSCs（GenBank ID） and their function** | | **Ref.** |
| --- | --- | --- | --- | --- | --- |
| *ZmOSC1* | OENC366048 | 61.35 | OM401325 | Hop-17(21)-en-3*β*-ol | [1] |
| *ZmOSC2* | OENC366049 | 77.81 | AK067451 | Isoarborinol synthase | [2] |
| *ZmOSC3* | OENC366050 | 64.39 | AK070534 | multifunctional synthase | [3] |
| *ZmOSC4* | OENC366051 | 77.98 | AK067451 | Isoarborinol synthase | [2] |
| *ZmOSC5* | OENC366052 | 61.42 | AK070534 | multifunctional synthase | [3] |
| *ZmOSC6* | OENC366053 | 63.83 | AK067451 | Isoarborinol synthase | [2] |
| *ZmOSC7* | OENC366054 | 87.22 | AK121211 | cycloartenol synthase | [3] |
| *ZmOSC8* | OENC366055 | 86.82 | AJ311790 | cycloartenol synthase | [4] |

**Table S2. Strains used in this study**

| **Strain** | **Description** | **Source/Ref.** |
| --- | --- | --- |
| *E. coli* strain | |  |
| BL21(DE3) | general expression host | Invitrogen |
| *S. cerevisiae* strains | |  |
| CEN.PK2-1C | general expression host | EUROSCARF |
| ZFctr | Triterpene Chassis | This study |
| ZF00 | ZFctr harboring plasmid pESC-dleu2 | This study |
| ZF01 | ZFctr harboring plasmid pESC-dleu2-ZmOSC1 | This study |
| ZF02 | ZFctr harboring plasmid pESC-dleu2-ZmOSC2 | This study |
| ZF03 | ZFctr harboring plasmid pESC-dleu2-ZmOSC3 | This study |
| ZF04 | ZFctr harboring plasmid pESC-dleu2-ZmOSC4 | This study |

**Table S3.** Primers used in this study

| **Primer** | **Sequences (5`-3`)** |
| --- | --- |
| ZmOSC1-F | ATGTGGAGGTTAAAGATCGGAGAG |
| ZmOSC1-R | TCAATTACTTTTGGTACATACTTTGCG |
| ZmOSC2-F | ATGTGGAGGCTGACGGTGGCCGAG |
| ZmOSC2-R | TCAATTGTCTTTCTTTGCACGAAGGCC |
| ZmOSC3-F | ATGTGGAGGCTAAAGATCGGCGAG |
| ZmOSC3-R | TTAACCTTTTTTTGCAACAAGTCGGCG |
| ZmOSC4-F | ATGTGGCGGCTGAAGATCGGCGAG |
| ZmOSC4-R | CTAGAAGGTTGGCCATTGGAGGTCACA |
| ZF01-F | AATATACCTCTATACTTTAACGTCAAGGAGAAAAAACCCATGTGGAGGTTAAAGATCGG |
| ZF01-R | TTTTCGGTTAGAGCGGATTTAATGATGATGATGATGATGTCAATTACTTTTGGTACATA |
| ZF02-F | AATATACCTCTATACTTTAACGTCAAGGAGAAAAAACCCATGTGGAGGCTGACGGTGGC |
| ZF02-R | TTTTCGGTTAGAGCGGATTTAATGATGATGATGATGATGTCAATTGTCTTTCTTTGCAC |
| ZF03-F | AATATACCTCTATACTTTAACGTCAAGGAGAAAAAACCCATGTGGAGGCTAAAGATCGG |
| ZF03-R | TTTTCGGTTAGAGCGGATTTAATGATGATGATGATGATGTTAACCTTTTTTTGCAACAA |
| ZF04-F | AATATACCTCTATACTTTAACGTCAAGGAGAAAAAACCCATGTGGCGGCTGAAGATCGG |
| ZF04-R | TTTTCGGTTAGAGCGGATTTAATGATGATGATGATGATGCTAGAAGGTTGGCCATTGGA |

**Table S4. Phylogenetic analysis of ZmOSCs from *Zea mays* and other oxidosqualene cyclases from diverse plant species**

| **OSC (GenBank ID)** | | | **Plant** | **Product** | **Ref.** |
| --- | --- | --- | --- | --- | --- |
| *SrBOS* | | AB455264 | *Stevia rebaudiana* | Baccharis oxide | [5] |
| *CpCPQ* | AB116238 | | *Cucurbita pepo* | Cucurbitadienol | [6] |
| *RcCAS* | DQ268870 | | *Ricinus communis* | Cycloartenol | [7] |
| *MiOSC1* | APG38074.1 | | *Maytenus ilicifolia* | Cycloartenol | [8] |
| *GgCAS1* | AB025968 | | *Glycyrrhiza glabra* | Cycloartenol | [9] |
| *CpCPX* | AB116237 | | *Cucurbita pepo* | Cycloartenol | [6] |
| *AtCAS1* | At2g07050 | | *Arabidopsis thaliana* | Cycloartenol | [10] |
| *RsCAS* | AB292608 | | *Rhizophora stylosa* | Cycloartenol | [11] |
| *PtCAS2* | ABX75048.2 | | *Polygala tenuifolia* | Cycloartenol | — |
| *PtCAS1* | ABX75046.1 | | *Polygala tenuifolia* | Cycloartenol | — |
| *PsCASPEA* | D89619 | | *Pisum sativum* | Cycloartenol | [12] |
| *PnCAS* | AB530328 | | *Polypodiodes niponica* | Cycloartenol | — |
| *PgPNX1* | AB009029 | | *Panax ginseng* | Cycloartenol | [13] |
| *OsOSC2* | AK121211 | | *Oryza sativa* | Cycloartenol | [3] |
| *LjOSC5* | AB181246 | | *Lotus japonicus* | Cycloartenol | [14] |
| *LcLCA* | BAU46473.1 | | *Lycopodium clavatum(3252)* | Cycloartenol | [15] |
| *LcCAS1* | AB033334 | | *Luffa cylindrica* | Cycloartenol | [16] |
| *KdCAS* | HM623872 | | *Kalanchoe daigremontiana* | Cycloartenol | [17] |
| *KcCAS* | AB292609 | | *Kandelia candel* | Cycloartenol | [11] |
| *DzCAS1* | AM697885 | | *Dioscorea zingiberensis* | Cycloartenol | — |
| *CsOSC1* | AB058507 | | *Costus speciosus* | Cycloartenol | [18] |
| *BpBPX2* | AB055510 | | *Betula platyphylla* | Cycloartenol | [19] |
| *BpBPX1* | AB055509 | | *Betula platyphylla* | Cycloartenol | [19] |
| *AmCAS1* | AF216755 | | *Abies magnifica* | Cycloartenol | — |
| *AcACX* | AB368375 | | *Adiantum capillus-veneris* | Cycloartenol | [20] |
| *AsCS1* | AJ311790 | | *Avena strigosa* | Cycloartenol^∗^ | [4] |
| *CrCAS1* | EDP09612 | | *Chlamydomonas reinhardtii* | Cycloartenol^∗^ | [21] |
| *DcDCD* | BAG68223.1 | | *Dryopteris crassirhizoma (Thick stemmed wood fern)* | Dammaradiene | [22] |
| *PgPNA* | AB265170 | | *Panax ginseng* | Dammarenediol II | [23] |
| *CaOSCCAS* | AAS01523.1 | | *Centella asiatica* | Dammarenediol-II | [24] |
| *MiOSC2* | APG38073.1 | | *Maytenus ilicifolia* | Friedelin | [8] |
| *KdFRS* | HM623870 | | *Kalanchoe daigremontiana* | Friedelin | [17] |
| *KdGLS* | HM623869 | | *Kalanchoe daigremontiana* | Glutinol | [17] |
| *OsIAS* | AK067451 | | *Oryza sativa* | Isoarborinol | [2] |
| *LcIMS1* | AB058643 | | *Luffa cylindrica* | Isomultiflorenol | [25] |
| *PgPNZ1* | AB009031 | | *Panax ginseng* | Lanosterol | [26] |
| *LjLAS* | AB244671 | | *Lotus japonicus* | Lanosterol | [27] |
| *AtLSS1* | At3g45130 | | *Arabidopsis thaliana* | Lanosterol | [28] |
| *RcLUS* | DQ268869 | | *Ricinus communis* | Lupeol | [7] |
| *LjOSC3* | AB181245 | | *Lotus japonicus* | Lupeol | [14] |
| *KdLUS* | HM623871 | | *Kalanchoe daigremontiana* | Lupeol | [17] |
| *GgLUS1* | AB116228 | | *Glycyrrhiza glabra* | Lupeol | [9] |
| *ToTRW* | AB025345 | | *Taraxacum officinale* | Lupeol | [29] |
| *OeOEW* | AB025343 | | *Olea europaea* | Lupeol | [29] |
| *BpBPW* | AB055511 | | *Betula platyphylla* | Lupeol | [19] |
| *BgLUS* | AB289586 | | *Bruguiera gymnorrhiza* | Lupeol | [11] |
| *AtMRN1* | At5g42600 | | *Arabidopsis thaliana* | Marneral | [30] |
| *KcMS* | AB257507 | | *Kandelia candel* | Mixed products^10^*^,^*^14^*^,^*^4^ | [31] |
| *LjAMY2* | AF478455 | | *Lotus japonicus* | Mixed products^10^*^,^*^4^*^,^*^UC^ | [32] |
| *CsOSC2* | AB058508 | | *Costus speciosus* | Mixed products^10^*^,^*^6^*^,^*^4^*^,^*^UC^ | [18] |
| *AtPEN6* | At1g78500 | | *Arabidopsis thaliana* | Mixed products^11^*^,^*^10^*^,^*^14^*^,^*^UC^ | [33] |
| *SlTTS2* | HQ266580 | | *Solanum lycopersicum* | Mixed products^14^*^,^*^4^*^,^*^23^ | [34] |
| *AtPEN1* | At4g15340 | | *Arabidopsis thaliana* | Mixed products^15^*^,^*^16^ | [35], [36] |
| *AtCAMS1* | At1g78955 | | *Arabidopsis thaliana* | Mixed products^2^*^,^*^3^*^,^*^4^ | [37] |
| *OsOSC8* | AK070534 | | *Oryza sativa* | Mixed products^22^*^,^*^UC^ | [3] |
| *RsM2* | AB263204 | | *Rhizophora stylosa* | Mixed products^24^*^,^*^4^*^,^*^10^ | [11] |
| *MdOSC3* | AMS36883.1 | | *Malus domestica* | Mixed products^4^*^,^*^10^ | [38] |
| *AtLUP1* | At1g78970 | | *Arabidopsis thaliana* | Mixed products^5^*^,^*^4^*^,^*^6^*^,^*^8^*^,^*^9^ | [39] |
| *RsM1* | AB263203 | | *Rhizophora stylosa* | Mixed products^6^*^,^*^4^*^,^*^10^ | [11] |
| *MdOSC2* | AMS36882.1 | | *Malus domestica* | Mixed products^6^*^,^*^4^*^,^*^10^ | [38] |
| *AtLUP5* | At1g66960 | | *Arabidopsis thaliana* | Mixed products^7^*^,^*^UC^ | [33] |
| *AtBARS1* | At4g15370 | | *Arabidopsis thaliana* | Mixed products^1+22other minor ones^ | [40] |
| *PsOSCPSM* | AB034803 | | *Pisum sativum* | Mixed products^14^*^,^*^4^*^,^*^23^*^,^*^9^*^,^*^12^*^,^*^10^*^,^*^6^*^,^*^8^ | [41] |
| *AtPEN3* | At5g36150 | | *Arabidopsis thaliana* | Mixed products^17^*^,^*^12^*^,^*^18^*^,^*^19^*^,^*^20^*^,^*^21^ | [42] |
| *MdOSC1* | ACM89977.1 | | *Malus domestica* | Mixed products^4,14^ | [43] |
| *AtLUP2* | At1g78960 | | *Arabidopsis thaliana* | Mixed products^4,8,7,10,11,12,13,9,14^ | [44] |
| *OsPS* | AK066327 | | *Oryza sativa* | Parkeol | [3] |
| *LcLCD* | BAU46472.1 | | *Lycopodium clavatum(3252)* | Pre-alpha-onocerin | [15] |
| *LcLCC* | BAU46471.1 | | *Lycopodium clavatum(3252)* | Pre-alpha-onocerin | [15] |
| *AtSHS1* | AB609123 | | *Aster tataricus* | Shionone | [45] |
| *KdTAS* | HM623868 | | *Kalanchoe daigremontiana* | Taraxerol | [17] |
| *AtTHAS1* | At5g48010 | | *Arabidopsis thaliana* | Thalianol | [46] |
| *OeOEA* | AB291240 | | *Olea europaea* | α-Amyrin | [47] |
| *SlTTS1* | HQ266579 | | *Solanum lycopersicum* | *β*-Amyrin | [34] |
| *MtAMYI* | AJ430607 | | *Medicago truncatula* | *β*-Amyrin | [32] |
| *AsbAS1* | AJ311789 | | *Avena strigosa* | *β*-Amyrin | [4] |
| *VhBS* | DQ915167 | | *Vaccaria hispanica* | *β*-Amyrin | [48] |
| *PtbAS* | EF107623 | | *Polygala tenuifolia* | *β*-Amyrin | — |
| *PsPSY* | AB034802 | | *Pisum sativum* | *β*-Amyrin | [12] |
| *PgPNY2* | AB014057 | | *Panax ginseng* | *β*-Amyrin | [13] |
| *PgPNY1* | AB009030 | | *Panax ginseng* | *β*-Amyrin | [13] |
| *LjAMY1* | AB181244 | | *Lotus japonicus* | *β*-Amyrin | [14] |
| *GgbAS1* | AB037203 | | *Glycyrrhiza glabra* | *β*-Amyrin | [49] |
| *EtAS* | AB206469 | | *Euphorbia tirucalli* | *β*-Amyrin | [50] |
| *BpBPY* | AB055512 | | *Betula platyphylla* | *β*-Amyrin | [19] |
| *BgbAS* | AB289585 | | *Bruguiera gymnorrhiza* | *β*-Amyrin | [31] |
| *AtLUP4* | At1g78950 | | *Arabidopsis thaliana* | *β*-Amyrin | [51] |
| *AsOXA1* | AY836006 | | *Aster sedifolius* | *β*-Amyrin | [52] |
| *AabAS* | EU330197 | | *Artemisia annua* | *β*-Amyrin | [53] |
| *AsHS1* | OM401324 | | *Avena strigosa* | Hopene B | [1] |
| *AsHS2* | OM401325 | | *Avena strigosa* | Hop-17(21)-en-3*β*-ol | [1] |
| *AcHS1* | OM401331 | | *Aquilegia coerulea* | Hopene B | [1] |

**Reference**

[1] Liang M, Zhang F, Xu J, Wang X, Wu R and Xue Z. A conserved mechanism affecting hydride shifting and deprotonation in the synthesis of hopane triterpenes as compositions of wax in oat. Proc Natl Acad Sci U S A 2022; 119: e2118709119. <https://doi.org/10.1073/pnas.2118709119>.

[2] Xue Z Y, Duan L X, Liu D, Guo J, Ge S, Dicks J, et al. Divergent evolution of oxidosqualene cyclases in plants. New Phytologist 2012; 193: 1022-38. <https://doi.org/10.1111/j.1469-8137.2011.03997.x>.

[3] Ito R, Mori K, Hashimoto I, Nakano C, Sato T and Hoshino T. Triterpene cyclases from oryza sativa l.: Cycloartenol, parkeol and achilleol b synthases. Organic Letters 2011; 13: 2678-81. <https://doi.org/10.1021/ol200777d>.

[4] Haralampidis K, Bryan G, Qi X, Papadopoulou K, Bakht S, Melton R, et al. A new class of oxidosqualene cyclases directs synthesis of antimicrobial phytoprotectants in monocots. Proc Natl Acad Sci U S A 2001; 98: 13431-6. <https://doi.org/10.1073/pnas.231324698>.

[5] Shibuya M, Sagara A, Saitoh A, Kushiro T and Ebizuka Y. Biosynthesis of baccharis oxide, a triterpene with a 3,10-oxide bridge in the a-ring. Org Lett 2008; 10: 5071-4. <https://doi.org/10.1021/ol802072y>.

[6] Shibuya M, Adachi S and Ebizuka Y. Cucurbitadienol synthase, the first committed enzyme for cucurbitacin biosynthesis, is a distinct enzyme from cycloartenol synthase for phytosterol biosynthesis. Tetrahedron 2004; 60: 6995-7003. <https://doi.org/10.1016/j.tet.2004.04.088>.

[7] Guhling O, Hobl B, Yeats T and Jetter R. Cloning and characterization of a lupeol synthase involved in the synthesis of epicuticular wax crystals on stem and hypocotyl surfaces of ricinus communis. Arch Biochem Biophys 2006; 448: 60-72. <https://doi.org/10.1016/j.abb.2005.12.013>.

[8] Souza-Moreira T M, Alves T B, Pinheiro K A, Felippe L G, De Lima G M A, Watanabe T F, et al. Friedelin synthase from maytenus ilicifolia: Leucine 482 plays an essential role in the production of the most rearranged pentacyclic triterpene. Sci Rep-Uk 2016; 6. <https://doi.org/ARTN> 36858

10.1038/srep36858.

[9] Hayashi H, Huang P, Takada S, Obinata M, Inoue K, Shibuya M, et al. Differential expression of three oxidosqualene cyclase mrnas in glycyrrhiza glabra. Biological & Pharmaceutical Bulletin 2004; 27: 1086-92. <https://doi.org/DOI> 10.1248/bpb.27.1086.

[10] Corey E J, Matsuda S P T and Bartel B. Isolation of an arabidopsis-thaliana gene encoding cycloartenol synthase by functional expression in a yeast mutant lacking lanosterol synthase by the use of a chromatographic screen. P Natl Acad Sci USA 1993; 90: 11628-32. <https://doi.org/DOI> 10.1073/pnas.90.24.11628.

[11] Basyuni M, Oku H, Tsujimoto E, Kinjo K, Baba S and Takara K. Triterpene synthases from the okinawan mangrove tribe, rhizophoraceae. Febs J 2007; 274: 5028-42. <https://doi.org/10.1111/j.1742-4658.2007.06025.x>.

[12] Morita M, Shibuya M, Lee M S, Sankawa U and Ebizuka Y. Molecular cloning of pea cdna encoding cycloartenol synthase and its functional expression in yeast. Biological & Pharmaceutical Bulletin 1997; 20: 770-5.

[13] Kushiro T, Shibuya M and Ebizuka Y. Beta-amyrin synthase - cloning of oxidosqualene cyclase that catalyzes the formation of the most popular triterpene among higher plants. Eur J Biochem 1998; 256: 238-44. <https://doi.org/DOI> 10.1046/j.1432-1327.1998.2560238.x.

[14] Sawai S, Shindo T, Sato S, Kaneko T, Tabata S, Ayabe S, et al. Functional and structural analysis of genes encoding oxidosqualene cyclases of lotus japonicus. Plant Sci 2006; 170: 247-57. <https://doi.org/10.1016/j.plantsci.2005.08.027>.

[15] Araki T, Saga Y, Marugami M, Otaka J, Araya H, Saito K, et al. Onocerin biosynthesis requires two highly dedicated triterpene cyclases in a fern lycopodium clavatum. Chembiochem 2016; 17: 288-90. <https://doi.org/10.1002/cbic.201500663>.

[16] Hayashi H, Hiraoka N, Ikeshiro Y, Yazaki K, Tanaka S, Kushiro T, et al. (1999) Molecular cloning of a cdna encoding cycloartenol synthase from luffa cylindrica (accession no. Ab033334). (pgr99-183). Plant physiology **121**, 1384-

[17] Wang Z H, Yeats T, Han H and Jetter R. Cloning and characterization of oxidosqualene cyclases from kalanchoe daigremontiana enzymes catalyzing up to 10 rearrangement steps yielding friedelin and other triterpenoids. Journal of Biological Chemistry 2010; 285: 29703-12. <https://doi.org/10.1074/jbc.M109.098871>.

[18] Kawano N, Ichinose K and Ebizuka Y. Molecular cloning and functional expression of cdnas encoding oxidosqualene cyclases from costus speciosus. Biological & Pharmaceutical Bulletin 2002; 25: 477-82. <https://doi.org/DOI> 10.1248/bpb.25.477.

[19] Zhang H, Shibuya M, Yokota S and Ebizuka Y. Oxidosqualene cyclases from cell suspension cultures of betula platyphylla var. Japonica: Molecular evolution of oxidosqualene cyclases in higher plants. Biological & Pharmaceutical Bulletin 2003; 26: 642-50.

[20] Shinozaki J, Shibuya M, Masuda K and Ebizuka Y. Squalene cyclase and oxidosqualene cyclase from a fern. Febs Lett 2008; 582: 310-8. <https://doi.org/10.1016/j.febslet.2007.12.023>.

[21] Merchant S S, Prochnik S E, Vallon O, Harris E H, Karpowicz S J, Witman G B, et al. The chlamydomonas genome reveals the evolution of key animal and plant functions. Science 2007; 318: 245-51. <https://doi.org/10.1126/science.1143609>.

[22] Shinozaki J, Shibuya M, Masuda K and Ebizuka Y. Dammaradiene synthase, a squalene cyclase, from dryopteris crassirhizoma nakai. Phytochemistry 2008; 69: 2559-64. <https://doi.org/10.1016/j.phytochem.2008.07.017>.

[23] Tansakul P, Shibuya M, Kushiro T and Ebizuka Y. Dammarenediol-ii synthase, the first dedicated enzyme for ginsenoside biosynthesis, in panax ginseng. Febs Lett 2006; 580: 5143-9. <https://doi.org/10.1016/j.febslet.2006.08.044>.

[24] Kim O T, Kim M Y, Huh S M, Bai D G, Ahn J C and Hwang B. Cloning of a cdna probably encoding oxidosqualene cyclase associated with asiaticoside biosynthesis from centella asiatica (l.) urban. Plant Cell Rep 2005; 24: 304-11. <https://doi.org/10.1007/s00299-005-0927-y>.

[25] Hayashi H, Huang P, Inoue K, Hiraoka N, Ikeshiro Y, Yazaki K, et al. Molecular cloning and characterization of isomultiflorenol synthase, a new triterpene synthase from luffa cylindrica, involved in biosynthesis of bryonolic acid. Eur J Biochem 2001; 268: 6311-7. <https://doi.org/DOI> 10.1046/j.0014-2956.2001.02588.x.

[26] Suzuki M, Xiang T, Ohyama K, Seki H, Saito K, Muranaka T, et al. Lanosterol synthase in dicotyledonous plants. Plant Cell Physiol 2006; 47: 565-71. <https://doi.org/10.1093/pcp/pcj031>.

[27] Sawai S, Akashi T, Sakurai N, Suzuki H, Shibata D, Ayabe S I, et al. Plant lanosterol synthase: Divergence of the sterol and triterpene biosynthetic pathways in eukaryotes. Plant Cell Physiol 2006; 47: 673-7. <https://doi.org/10.1093/pcp/pcj032>.

[28] Kolesnikova M D, Xiong Q B, Lodeiro S, Hua L and Matsuda S P T. Lanosterol biosynthesis in plants. Arch Biochem Biophys 2006; 447: 87-95. <https://doi.org/10.1016/j.abb.2005.12.010>.

[29] Shibuya M, Zhang H, Endo A, Shishikura K, Kushiro T and Ebizuka Y. Two branches of the lupeol synthase gene in the molecular evolution of plant oxidosqualene cyclases. Eur J Biochem 1999; 266: 302-7. <https://doi.org/DOI> 10.1046/j.1432-1327.1999.00875.x.

[30] Xiong Q B, Wilson W K and Matsuda S P T. An arabidopsis oxidosqualene cyclase catalyzes iridal skeleton formation by grob fragmentation. Angew Chem Int Edit 2006; 45: 1285-8. <https://doi.org/10.1002/anie.200503420>.

[31] Basyuni M, Oku H, Inafuku M, Baba S, Iwasaki H, Oshiro K, et al. Molecular cloning and functional expression of a multifunctional triterpene synthase cdna from a mangrove species kandelia candel (l.) druce. Phytochemistry 2006; 67: 2517-24. <https://doi.org/10.1016/j.phytochem.2006.09.016>.

[32] Iturbe-Ormaetxe I, Haralampidis K, Papadopoulou K and Osbourn A E. Molecular cloning and characterization of triterpene synthases from medicago truncatula and lotus japonicus. Plant Mol Biol 2003; 51: 731-43. <https://doi.org/10.1023/a:1022519709298>.

[33] Ebizuka Y, Katsube Y, Tsutsumi T, Kushiro T and Shibuya M. Functional genomics approach to the study of triterpene biosynthesis. Pure Appl Chem 2003; 75: 369-74. <https://doi.org/DOI> 10.1351/pac200375020369.

[34] Wang Z H, Guhling O, Yao R N, Li F L, Yeats T H, Rose J K C, et al. Two oxidosqualene cyclases responsible for biosynthesis of tomato fruit cuticular triterpenoids. Plant Physiology 2011; 155: 540-52. <https://doi.org/10.1104/pp.110.162883>.

[35] Kolesnikova M D, Obermeyer A C, Wilson W K, Lynch D A, Xiong Q B and Matsuda S P T. Stereochemistry of water addition in triterpene synthesis: The structure of arabidiol. Organic Letters 2007; 9: 2183-6. <https://doi.org/10.1021/ol070709b>.

[36] Xiang T, Shibuya M, Katsube Y, Tsutsumi T, Otsuka M, Zhang H, et al. A new triterpene synthase from arabidopsis thaliana produces a tricyclic triterpene with two hydroxyl groups. Organic Letters 2006; 8: 2835-8. <https://doi.org/10.1021/ol060973p>.

[37] Kolesnikova M D, Wilson W K, Lynch D A, Obermeyer A C and Matsuda S P. Arabidopsis camelliol c synthase evolved from enzymes that make pentacycles. Org Lett 2007; 9: 5223-6. <https://doi.org/10.1021/ol702399g>.

[38] Andre C M, Legay S, Deleruelle A, Nieuwenhuizen N, Punter M, Brendolise C, et al. Multifunctional oxidosqualene cyclases and cytochrome p450 involved in the biosynthesis of apple fruit triterpenic acids. New Phytologist 2016; 211: 1279-94. <https://doi.org/10.1111/nph.13996>.

[39] Herrera J B R, Bartel B, Wilson W K and Matsuda S P T. Cloning and characterization of the arabidopsis thaliana lupeol synthase gene. Phytochemistry 1998; 49: 1905-11.

[40] Lodeiro S, Xiong Q B, Wilson W K, Kolesnikova M D, Onak C S and Matsuda S P T. An oxidosqualene cyclase makes numerous products by diverse mechanisms: A challenge to prevailing concepts of triterpene biosynthesis. J Am Chem Soc 2007; 129: 11213-22. <https://doi.org/10.1021/ja073133u>.

[41] Morita M, Shibuya M, Kushiro T, Masuda K and Ebizuka Y. Molecular cloning and functional expression of triterpene synthases from pea (pisum sativum) new alpha-amyrin-producing enzyme is a multifunctional triterpene synthase. Eur J Biochem 2000; 267: 3453-60. <https://doi.org/10.1046/j.1432-1327.2000.01357.x>.

[42] Morlacchi P, Wilson W K, Xiong Q B, Bhaduri A, Sttivend D, Kolesnikova M D, et al. Product profile of pen3: The last unexamined oxidosqualene cyclase in arabidopsis thaliana. Organic Letters 2009; 11: 2627-30. <https://doi.org/10.1021/ol9005745>.

[43] Brendolise C, Yauk Y K, Eberhard E D, Wang M, Chagne D, Andre C, et al. An unusual plant triterpene synthase with predominant alpha-amyrin-producing activity identified by characterizing oxidosqualene cyclases from malus x domestica. Febs J 2011; 278: 2485-99. <https://doi.org/10.1111/j.1742-4658.2011.08175.x>.

[44] Kushiro T, Shibuya M, Masuda K and Ebizuka Y. A novel multifunctional triterpene synthase from arabidopsis thaliana. Tetrahedron Lett 2000; 41: 7705-10. <https://doi.org/Doi> 10.1016/S0040-4039(00)01347-2.

[45] Sawai S, Uchiyama H, Mizuno S, Aoki T, Akashi T, Ayabe S, et al. Molecular characterization of an oxidosqualene cyclase that yields shionone, a unique tetracyclic triterpene ketone of aster tataricus. Febs Lett 2011; 585: 1031-6. <https://doi.org/10.1016/j.febslet.2011.02.037>.

[46] Fazio G C, Xu R and Matsuda S P T. Genome mining to identify new plant triterpenoids. J Am Chem Soc 2004; 126: 5678-9. <https://doi.org/10.1021/ja0318784>.

[47] Saimaru H, Orihara Y, Tansakul P, Kang Y H, Shibuya M and Ebizuka Y. Production of triterpene acids by cell suspension cultures of olea europaea. Chem Pharm Bull 2007; 55: 784-8. <https://doi.org/DOI> 10.1248/cpb.55.784.

[48] Meesapyodsuk D, Balsevich J, Reed D W and Covello P S. Saponin biosynthesis in saponaria vaccaria. Cdnas encoding beta-amyrin synthase and a triterpene carboxylic acid glucosyltransferase. Plant Physiol 2007; 143: 959-69. <https://doi.org/10.1104/pp.106.088484>.

[49] Hayashi H, Huang P Y, Kirakosyan A, Inoue K, Hiraoka N, Ikeshiro Y, et al. Cloning and characterization of a cdna encoding beta-amyrin synthase involved in glycyrrhizin and soyasaponin biosyntheses in licorice. Biological & Pharmaceutical Bulletin 2001; 24: 912-6. <https://doi.org/DOI> 10.1248/bpb.24.912.

[50] Kajikawa M, Yamato K T, Fukuzawa H, Sakai Y, Uchida H and Ohyama K. Cloning and characterization of a cdna encoding beta-amyrin synthase from petroleum plant euphorbia tirucalli l. Phytochemistry 2005; 66: 1759-66. <https://doi.org/10.1016/j.phytochem.2005.05.021>.

[51] Shibuya M, Katsube Y, Otsuka M, Zhang H, Tansakul P, Xiang T, et al. Identification of a product specific beta-amyrin synthase from arabidopsis thaliana. Plant Physiol Bioch 2009; 47: 26-30. <https://doi.org/10.1016/j.plaphy.2008.09.007>.

[52] Cammareri M, Consiglio M F, Pecchia P, Corea G, Lanzotti V, Ibeas J I, et al. Molecular characterization of beta-amyrin synthase from aster sedifolius l. And triterpenoid saponin analysis. Plant Sci 2008; 175: 255-61. <https://doi.org/10.1016/j.plantsci.2008.04.004>.

[53] Kirby J, Romanini D W, Paradise E M and Keasling J D. Engineering triterpene production in saccharomyces cerevisiae-beta-amyrin synthase from artemisia annua. Febs J 2008; 275: 1852-9. <https://doi.org/10.1111/j.1742-4658.2008.06343.x>.
